# Supplementary material for: Stage-dependent remodeling of projections to motor cortex in ALS mouse model revealed by a new variant retrograde-AAV9
Source: eLife. 2018 Aug 23;7:e36892. doi: 10.7554/eLife.36892 (PMC6125125; doi:10.7554/eLife.36892)
Supplement: Transparent reporting form [file elife-36892-transrepform.docx]

***eLife’s* transparent reporting form**

We encourage authors to provide detailed information *within their submission* to facilitate the interpretation and replication of experiments. Authors can upload supporting documentation to indicate the use of appropriate reporting guidelines for health-related research (see [EQUATOR Network](http://www.equator-network.org/%20)), life science research (see the [BioSharing Information Resource](https://biosharing.org/)), or the [ARRIVE guidelines](http://www.plosbiology.org/article/info:doi/10.1371/journal.pbio.1000412) for reporting work involving animal research. Where applicable, authors should refer to any relevant reporting standards documents in this form.

If you have any questions, please consult our Journal Policies and/or contact us: [editorial@elifesciences.org](mailto:editorial@elifesciences.org).

**Sample-size estimation**

- You should state whether an appropriate sample size was computed when the study was being designed
- You should state the statistical method of sample size computation and any required assumptions
- If no explicit power analysis was used, you should describe how you decided what sample (replicate) size (number) to use

Please outline where this information can be found within the submission (e.g., sections or figure legends), or explain why this information doesn’t apply to your submission:

We have reported the choices made for sample size calculations in the “Animals” paragraph of the methods section, reading as “In order to maintain the data analysis tractable and in absence of previous data on quantitative analysis of input connectivity in ALS mice, we estimated the sample size based on the mean number and standard deviation of thalamic neurons at P90, anticipating a genotype effect of at least 30-40%, with α=0.05 and 90% power, resulting in group size between 3 and 5”. We are aware that these settings may have led to a false negative detection in structures with comparatively low number of neurons and high variations; however, the need to maintain the project experimentally tractable was balanced with the detection of smaller genotype effects.

**Replicates**

- You should report how often each experiment was performed
- You should include a definition of biological versus technical replication
- The data obtained should be provided and sufficient information should be provided to indicate the number of independent biological and/or technical replicates
- If you encountered any outliers, you should describe how these were handled
- Criteria for exclusion/inclusion of data should be clearly stated
- High-throughput sequence data should be uploaded before submission, with a private link for reviewers provided (these are available from both GEO and ArrayExpress)

Please outline where this information can be found within the submission (e.g., sections or figure legends), or explain why this information doesn’t apply to your submission:

Each animal is considered a biological replicate, and because of the nature of the readout (neuronal counting), it is considered a single technical replicate. For experiments in which sampling was obtained from multiple sections from each animal, or from multiple neurons from a given animal, we indicate the number of sections and the number of neurons/animal used.

**Statistical reporting**

- Statistical analysis methods should be described and justified
- Raw data should be presented in figures whenever informative to do so (typically when N per group is less than 10)
- For each experiment, you should identify the statistical tests used, exact values of N, definitions of center, methods of multiple test correction, and dispersion and precision measures (e.g., mean, median, SD, SEM, confidence intervals; and, for the major substantive results, a measure of effect size (e.g., Pearson's r, Cohen's d)
- Report exact p-values wherever possible alongside the summary statistics and 95% confidence intervals. These should be reported for all key questions and not only when the p-value is less than 0.05.

Please outline where this information can be found within the submission (e.g., sections or figure legends), or explain why this information doesn’t apply to your submission:

- Statistical methods applied for mouse data are reported in the paragraph named “*Statistical analysis- mouse experiments*” in the methods section. The analysis of fMRI data is reported extensively in the methods section under the paragraphs: “*Preprocessing of functional data” “Functional data analysis” “Functional connectome reconstruction” “Interference statistics”*

*-*Because of the large number of groups, due to the extensive anatomical classification used, we have decided to report the data in histogram format in the majority of the graphs, to improve readability; we provide individual data whenever possible, e.g. for spine density analysis. We have nevertheless provided the numerical data associated with each figure as supplementary information

- in the figure legend of each figure, and again in the main text, we indicate the statistical test used, the N value and the precise p value.

(For large datasets, or papers with a very large number of statistical tests, you may upload a single table file with tests, Ns, etc., with reference to sections in the manuscript.)

**Group allocation**

- Indicate how samples were allocated into experimental groups (in the case of clinical studies, please specify allocation to treatment method); if randomization was used, please also state if restricted randomization was applied
- Indicate if masking was used during group allocation, data collection and/or data analysis

Please outline where this information can be found within the submission (e.g., sections or figure legends), or explain why this information doesn’t apply to your submission:

Since all littermates from a given litter were used for experimental purpose, no randomization was applied; however, the genotype of the animals was not revealed to the experimenter till the completion of the analysis. We report this information in the methods section (paragraph “animals”) with the following sentence: “All male mice from a given litter were employed and the experimenter was kept blind to their genotype till the data analysis stage”.

**Additional data files (“source data”)**

- We encourage you to upload relevant additional data files, such as numerical data that are represented as a graph in a figure, or as a summary table
- Where provided, these should be in the most useful format, and they can be uploaded as “Source data” files linked to a main figure or table
- Include model definition files including the full list of parameters used
- Include code used for data analysis (e.g., R, MatLab)
- Avoid stating that data files are “available upon request”

Please indicate the figures or tables for which source data files have been provided:

Figure 1, 2,3,4,5,6,8
